# The Indian Medical Service
*"The Indian Medical Service: A Guide for Intending Candidates for Commissions, and for the Junior Officers of the Service." By W. W. Webb, M.B., Surgeon Bengal Army. (London: Messrs. W. Thacker and Co., 87, Newgate-street.)


**Published:** 1891-06-13

**Authors:** 


### THE INDIAN MEDICAL SERVICE.*

The author explains that when he had completed his curriculum he desired reliable information concerning the Indian medical service, but was unable to obtain any details. The memorandum issued to applicants by the India Office relates only to the military part of the service, and information from retired officers was of too general a nature to be of much assistance in enabling a man to decide for or against the service as a career. On that account, therefore, Dr. Webb has published the book under review. It is on the lines of several books which have been previously published, notably, McCosh's "Advice to Officers in India," now some forty years old; and Francis's "Medical Officers' *Vade Mecum*," published some twenty years back. First Dr. Webb gives some professional advice to intending candidates, mentioning that they should pay special attention to diseases of the eye, calculus of the bladder, bowel disorders, and midwifery, all being conditions which the Indian medical officer is constantly meeting with. Next there are hints about the professional examination for entrance into the service; also regarding the course of instruction given to successful candidates at Netley. Then follow observations about outfit, and hints which may be found useful on arrival in India. The aspirant will do well to follow the hints on outfit, as he will not then be burthened with the ninety-nine unnecessary and expensive articles outfitters delight in selling. The arrival hints are also very good advice, and would certainly prove useful to the neophyte in a strange land. But one item we certainly do not agree with. "Officers proceeding up country," Dr. Webb says, "will do well to provide themselves with good solar topis (helmets) before leaving Bombay." In our opinion officers should take solar helmets from England, and wear them too, if they go ashore in the daytime at Port Said or at Suez. They should also wear them in the Red Sea, and if they go ashore at Aden, and they should land with their solar topis on in Bombay. Many a fever has been contracted from heedless exposure to the sun before reaching India. An omission is that the author says nothing about engaging servants on arrival in India; and he does not caution against those men who, under the guise of servants, join a new arrival for a few days, and then desert at the most inopportune moment. The grades and precedence of medical officers are then given from the Surgeon.General, ranking as a Major-General, to the Surgeon, ranking as a Captain. The pay and pension are also shown. Then there are details regarding the medical services of the three Presidencies (Bengal, Bombay, and Madras), showing the number of officers in military employ, and the number in civil employ. For it should be known that, although medical officers of the Indian service are members of a military service, and therefore belong to the army, a large number are employed in a civil capacity, from which, however, they are liable to be recalled at any time, should war or other emergency demand their services. Unless, however, an officer is appointed to a large civil station or becomes a professor at one of the colleges, it may be questioned if altogether he is not better off in the military branch of the service. At some stations where political officers are located, and to which medical officers are attached, Dr. Webb says there is considerable private practice among rich native communities, and he instances Hyderabad, Baghdad, Indore, and Bikanir. As the author was surgeon at Bikanir it may be presumed this is correct. But in former days there was no private practice in the desert city of Bikanir.

We are certainly able to recommend Dr. Webb's book to those aspirants seeking to obtain reliable information concerning the Indian Medical Service, but it is not adapted for men already in the service. Officers are obliged by the regulations to possess themselves of the sanctioned "Army Regulations for India," the last edition being published in 1889. In this book, every rule and regulation affecting the medical officer in military employ will be found. Officers who obtain civil employ must be provided with "The Civil Service Regulations," embodying the civil pay code, the acting allowance code, and the leave and travelling allowance code, the last edition also published in 1889. These are the books by which the medical officer must be guided, and which he must quote, chapter and verse, in any official correspondence. A medical officer will not be long in the service before he finds it necessary to appeal against, or to dispute something or other—probably some question affecting pay, or acting allowance, or travelling allowance. And it would be useless quoting Dr. Webb's book either to the Controller-General of the Military Department, or to the Accountant-General of the Civil Department. He must base his objections or demands on the authoritative rules and regulations mentioned above. It is astonishing, when, in these books, everything seems to be plainly laid down, how many quibbles arise, and how differently officers interpret rules and regulations to the meaning given to them by the financial authorities. The former, of course, are biassed by their own interests, the latter endeavour to save the public exchequer, and officers not unfrequently find that what they thought implied one thing, really means something else. We therefore say, that as soon as a medical officer is located in India, he should begin to study the Army Regulations and the Civil Code, not only with reference to pay and allowance, but also to enable him to meet demands on fifty different matters which may crop up at any time, and which he can only dispose of in accordance with the regulations we have named. Dr. Webb's book is susceptible of considerable improvement. In the first place, it should be interleaved blank, for old regulations are constantly being changed, and new ones are constantly being framed, and a blank leaf would allow of such orders being inserted in their proper places. Again, nearly every sentence in Dr. Webb's book, after the first few pages, is based on some order which has been issued by the Government, or by the Commander-in-Chief. If the number, date, and department of such orders were given in the margin, the value of the book would be immensely increased. Of course, this would entail some trouble, and might possibly add a little to the cost of printing, but, on the other hand, it would render the book a handy one of reference, when the more bulky official Army Regulations and Civil Regulations are not at hand, and so economise space in the cabin, the bullock trunk, and the tent. With these two alterations, the present germ might expand into a really useful book for all grades of medical officers.

* "The Indian Medical Service: A Guide for Intending Candidates for Commissions, and for the Junior Officers of the Service." By W. W. Webb, M.B., Surgeon Bengal Army. (London: Messrs. W. Thacker and Co., 87, Newgate-street.)

